# Root canal therapy used in conjunction with follow‐ups for the nonsurgical handling of an enormous periapical lesion of endodontic origin: Clinical case record

**DOI:** 10.1002/ccr3.9074

**Published:** 2024-06-10

**Authors:** Nitin R. Rao, Abdul Mujeeb, Aishwarya A. Kottur, Ankita Mathur, Niher Tabassum Siddiqua Snigdha, Mohmed Isaqali Karobari

**Affiliations:** ^1^ Department of Conservative Dentistry and Endodontics S.J.M. Dental College and Hospital Chitradurga Karnataka India; ^2^ Department of Periodontology Dr. D. Y. Patil Dental College and Hospital, Dr. D. Y. Patil Vidyapeeth Pune India; ^3^ Department of Dental Research, CGHR, Saveetha Medical College and Hospitals Saveetha Institute of Medical and Technical Sciences Chennai Tamil Nadu India; ^4^ Department of Restorative Dentistry & Endodontics, Faculty of Dentistry University of Puthisastra Phnom Penh Cambodia

**Keywords:** dentistry, endodontic, healing, nonsurgical, periapical lesion, root canal treatment

## Abstract

**Key Clinical Message:**

The main objective of root canal therapy is to locate all the canals, cleaning, and shaping, and obturation to obtain fluid tight seal and to heal the periapical lesion if present.

**Abstract:**

The proper cleaning, shaping, and disinfection of the pulp chambers, as well as the filling of the canals, are critical to the efficacy of treatment with root canals. The success of an endodontically treated tooth is dependent on the accuracy of the diagnosis, disinfection, cleaning and shaping, obturation, and finally, the prosthetic rehabilitation management. Root canal therapy should provide a hermatic as well as fluid impenetrable seal which prevents the progression of periapical infection. There are two ways to treat such lesions: surgical and nonsurgical methods. If the root canal is cleaned, shaped, and sealed properly and adequately without the use of a surgical procedure, these lesions will recover during nonsurgical root canal therapy. This case series focuses primarily on the nonsurgical treatment of an enormous periapical lesion and provides evidence that these lesions respond well without surgery.

## 1 INTRODUCTION

Pulp is a mass of connective tissue that resides within the center of the tooth, directly beneath the layer of dentin and also secured by enamel, dentin, cementum. The majority of pain‐related impulses originate in the pulp chamber, which is intensely supplied with blood and is the stimulated part of the teeth. Notable injury to such vascularized structure of tooth has a significant role in inflammation process which could result in pulp death if left untreated.^1^ Possible conditions that causes pulp necrosis include dental decay, injury, tooth wear or unsatisfactory therapy of root canals.^1^, ^2^

The necrosed pulp is extremely prone to establishment of the microbes present in a mouth's cavity.^3^ The microorganisms along with other components of the cell activates an inflammatory process in the periapical region of the tooth.^3^ Eventually an immunopathological process give rise to periapical infections.^3^ The beginning and progression of periradicular lesions are significantly influenced by microbes and the products they produce.^1^ Time when the root canal infection has been developed both host's immune system and antibiotics fail to work in limiting the infection brought on by a reduction in circulation.^1^ These infections or lesions come into view during radiographic examination or by patient's complain on pain and discomfort.^3^

The aim of endodontic therapy is to bring back the affected tooth to a normal healthy form with no use of any surgical methods. These lesions in the periapical region have to be first managed nonsurgically. Only when the nonsurgical treatment fails, surgical procedure should be considered. A rate of efficacy of 85% can be achieved without surgery for these periapical infections. Following nonsurgical endodontic treatment, the rate of full and limited repair of lesions in the periapical region is around 94.4%.^3^

Calcium hydroxide is one of the most common intracanal medication. Its widespread and diverse features, such as antibacterial property, tissue disintegrating capacity, and the capacity to induce healing via hard tissue synthesis, make it widely acceptable for therapy. Calcium hydroxide delivers a highly alkaline condition having a pH around 12. This environment makes most of the microorganisms unable to survive.^3^

Metapex is one of the most commonly used and readily available calcium hydroxide that has been combined with silicon‐oil‐iodoform paste. It is a nontoxic paste including a combination of calcium hydroxide (30%), silicone oil (22.4%), and iodoform (40.4%). It is used with the help of syringe and disposable tips. Clinically it is used for apexification/apexogenesis procedures.^4^

This article's objective was to describe a nonsurgical approach of large periapical lesion of endodontic origin by root canal therapy with follow‐ups.

## 2 CASE HISTORY/EXAMINATION

**FIGURE 1:**
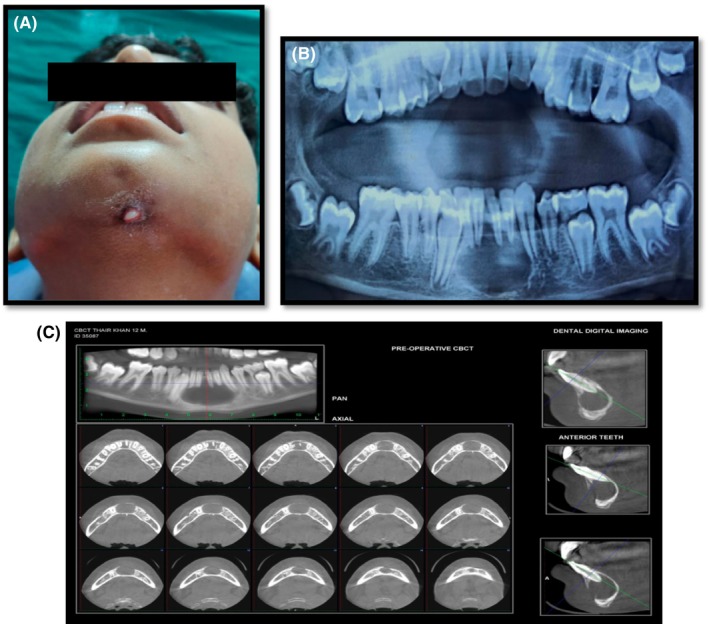
(A) Preoperative extraoral picture. (B) Preoperative radiograph.(C) Preoperative CBCT.

The Department of Conservative Dentistry and Endodontics at our institute received a visit from a 12‐year‐old male patient, whose main complaint was purulent discharge under his chin area. A multifocal dermal lesion was discovered in the middle region of his chin during extraoral inspection (Figure 1A). The growth had a purulent discharge coming from it and was mushy to the touch. According to the condition's past, the growth had alternately appeared and vanished during the previous 6 months. Patient neglected this issue for the previous 6 months before reporting it to the department out of aesthetic concerns.

Neither caries nor discomfort upon percussion in the mandibular anterior teeth were seen during the intraoral inspection. There was the presence of Elli's class II fracture with respect to tooth 31, which was non‐tender on percussion. On pulp vitality testing, teeth 41, 42, 31, 32, 33 were not responsive to the thermal test or the electric pulp test, but a radiographic scan identified the same set of teeth as having definite periapical radiolucency (Figure 1B,C). But tooth 43 showed normal positive response to vitality test. The patient revealed an incident of trauma to the jaw area 2 years prior during case history interview.

Morphological and radiographic studies led to the diagnosis of extraoral sinus and chronic suppurative periapical periodontitis in connection with teeth 41, 42, 31, 32, 33. So the treatment planned for the teeth 41, 42, 31, 32, 33 was a nonsurgical endodontic treatment under local anesthesia.

## 3 METHODS

**FIGURE 2:**
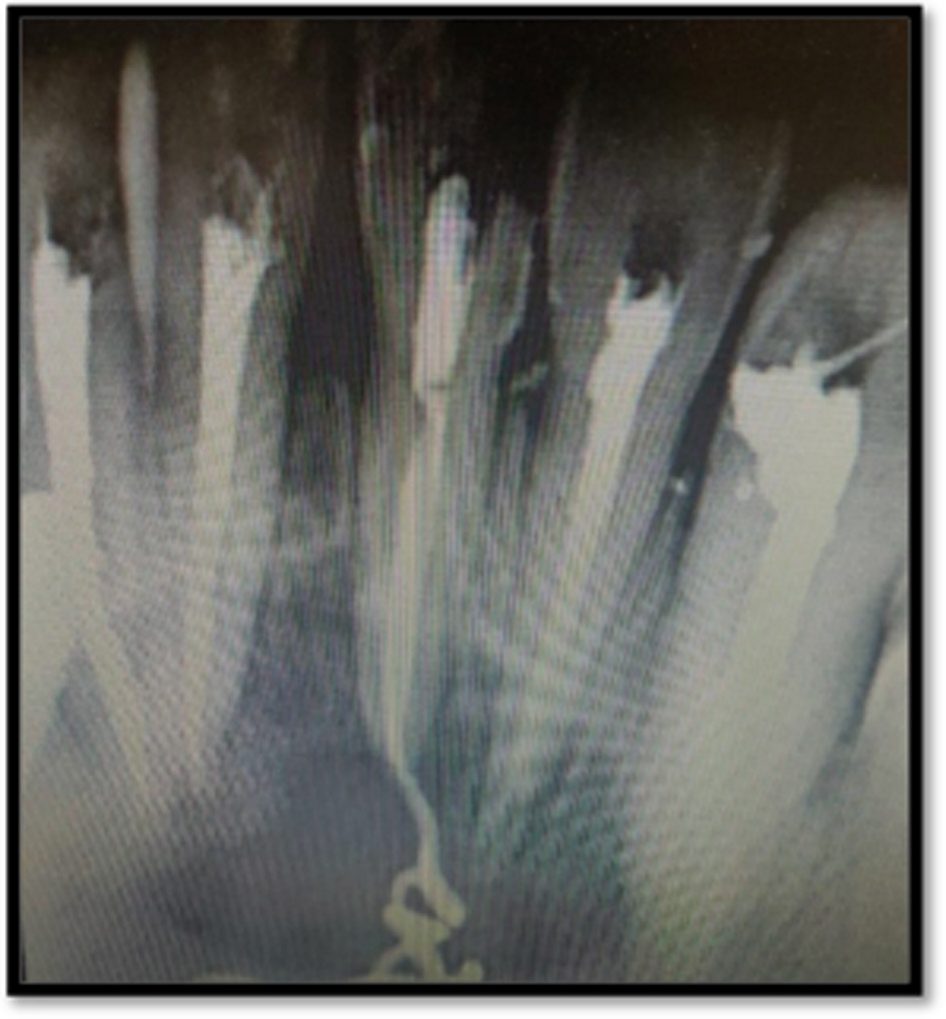
Intracanal medicament placed.

**FIGURE 3:**
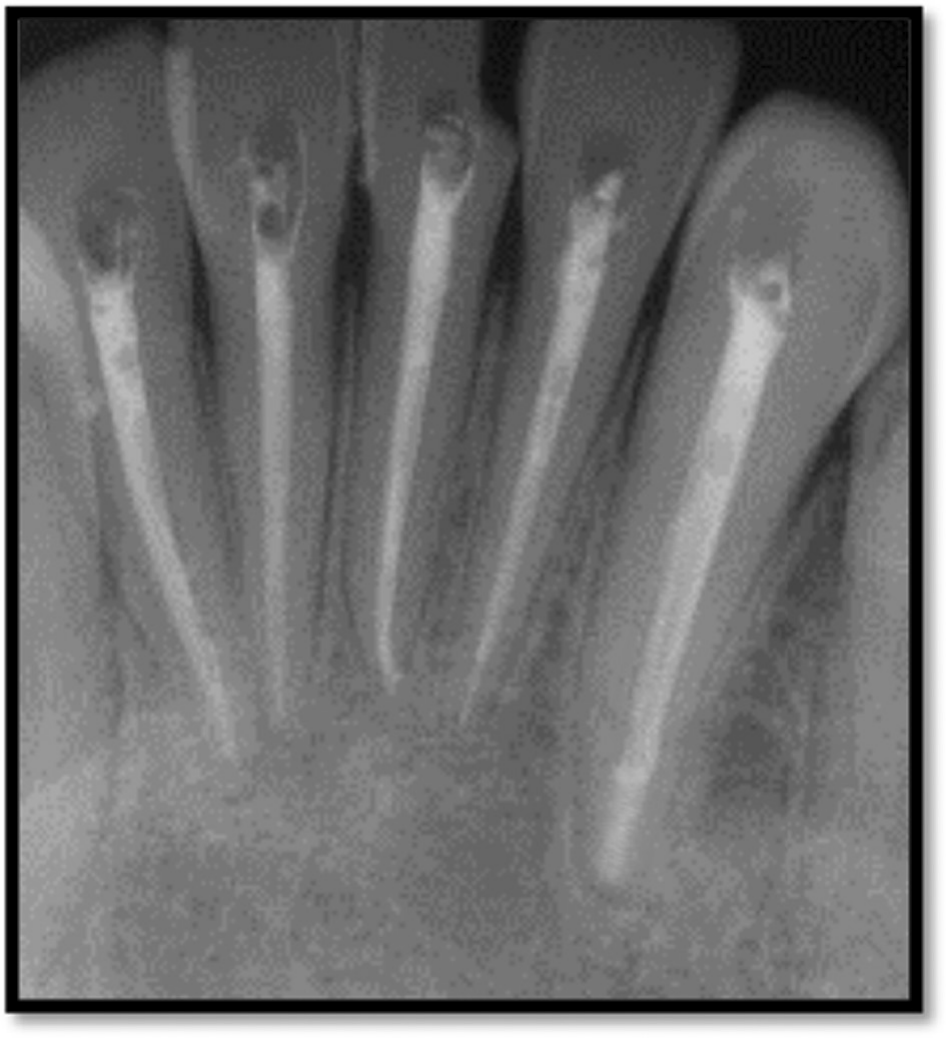
Fifteen‐month follow‐up radiograph post‐obturation.

After taking informed consent from the patient, local anesthesia was administered using lidocaine 2% with adrenaline 1:80,000. After that with rubber dam access opening was done with respect to teeth 41, 42, 31, 32, 33. A periapical radiography and the Root ZX small electronic apex locator (J. Morita, Kyoto, Japan) were used to establish the working length. Cleaning and shaping of the root canal network was done to size F4 (Dentsply Protaper Gold files). 5.25% Sodium hypochlorite (Nice Chemical PVT Ltd, Kochi, India) was activated using Endoactivator followed by 17% ethylenediaminetetraacetic acid (Nice Chemical PVT Ltd, Kochi, India) for 1 min per canal. Normal saline (Infutec Healthcare Limited, Indore, Madhya Pradesh, India) was used as the final flush. The canals were dried using paper points. Calcium hydroxide with iodoform was administered for a week as an intracanal medication (Figure 2). The extraoral lesion showed signs of recovery after a week and no pus‐like discharge was present. After 1 month for monitoring, the patient was contacted and the intracanal medication was changed. Canals were then obturated with the aid of gutta percha and AH plus sealer (Figure 3). The CBCT images were assessed and diagnosed as a radicular cyst in the anterior region by the consultant maxillofacial radiologist. After 3 months on radiographic examination showed bony healing changes were seen.

## 4 CONCLUSION

In the present case report, endodontic approach without surgery using the correct cleaning, decontamination, filling of the root canal framework, and administration of intracanal medication such calcium hydroxide produced beneficial outcomes in aiding the recovery of lesions in the periapical region. No matter how big the area of damage is, endodontic therapy without surgery should always be tried for managing periapical lesions. Also it is necessary to have a follow‐ups for better prognosis.

## 5 RESULTS AND FOLLOW‐UPS

**FIGURE 4:**
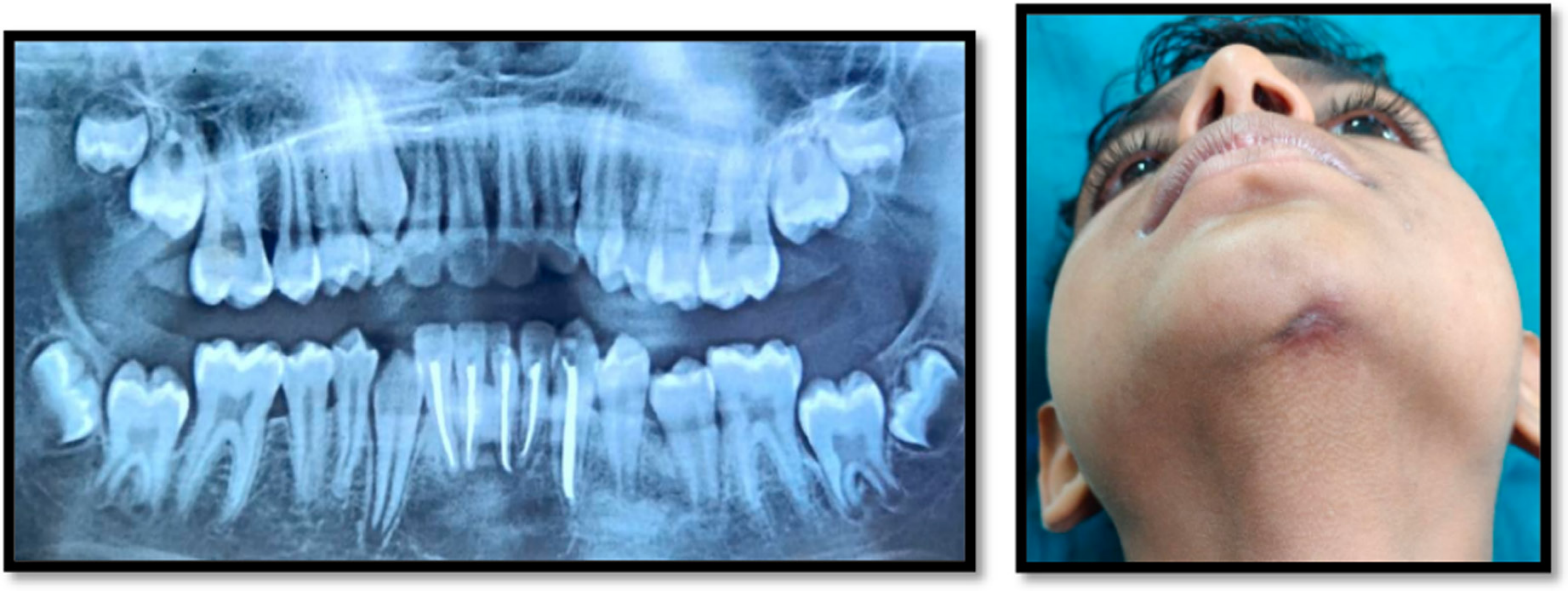
Three‐month follow‐up.

**FIGURE 5:**
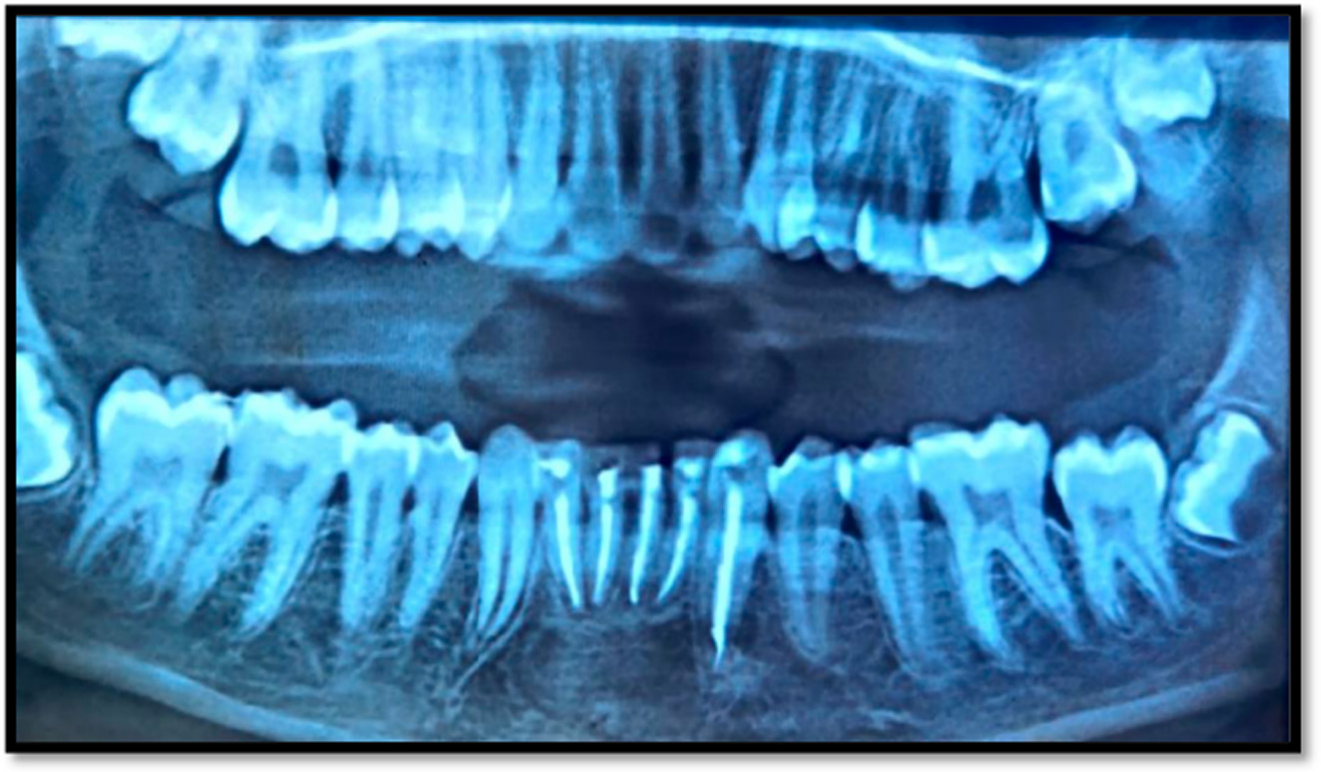
One‐year follow‐up.

**FIGURE 6:**
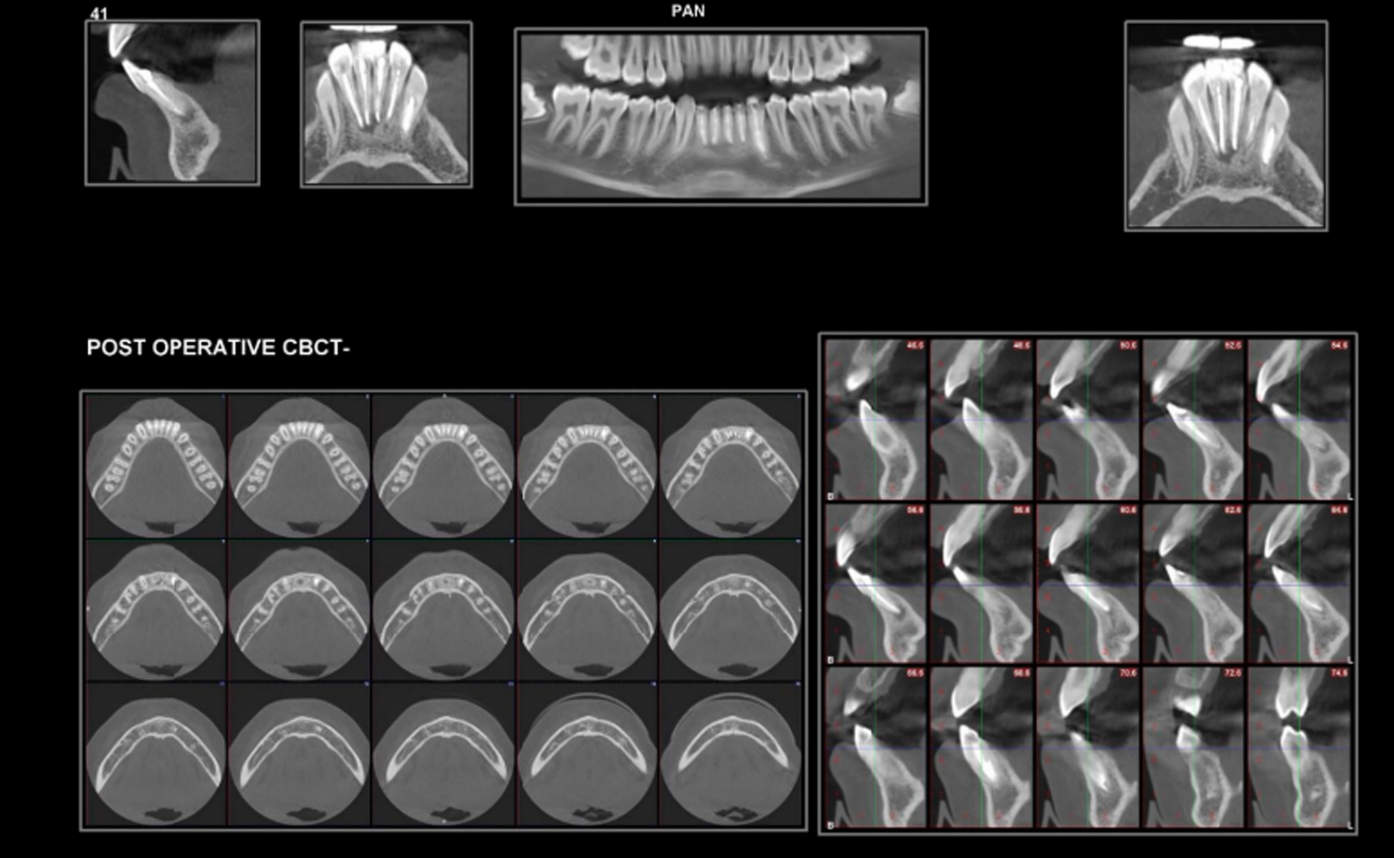
Two‐year follow‐up—CBCT.

After 3 months, the patient was discovered to be symptom‐free, the extraoral lesion had subsided, and the radiographic evaluation revealed that the periapical pathology with regard to the patient's teeth 41, 42, 31, 32, 33 had healed (Figure 4). Later patient was recalled after 1 and 2 years for follow‐up (Figures 5 and 6).

## 6 DISCUSSION

The main objective of endodontic treatment is to eradicate microbes to the maximum from root canal complex. Once the pulp becomes necrotic, its environment is suitable for the microorganism present in the root canal spaces to multiply and to instigate an inflammatory reaction causing the development of periapical lesion. The therapy regimen ranges from endodontic therapy without surgery and/or surgical intervention for the management of periapical lesions.^3^, ^5^

Nonsurgical endodontic therapy should always be considered for the management of periapical lesions when compared to surgical intervention as most of the patients are mentally more concerned about surgical approach. Also awareness of the risk factors and complications associated with medically compromised patients during surgical approach needs to be considered during surgical intervention.^6^

The cause of the extraoral sinus tract comprises pulpal conditions, lingering long‐term infections of the jaw, and dental trauma. Via periapical area, the pus that comes from these pathways travels in the direction of least resistance. In accordance with where the muscle connections and facial sheaths are located, the sinus tract emerges from the cortical plate and becomes an intraoral or extraoral sinus.^2^, ^7^

Usually these sinus tracts required surgical management rather than nonsurgical approach because it was once believed that epithelium lined them. Various studies revealed that they were not bordered with epithelium, but rather with granulation tissue. So nonsurgical endodontic management can be considered for these sinus tracts.^2^

Endodontic procedure primary objective is to purge germs from the root canal regions. The removal of microorganisms from the root canal network is aided by irrigation and intra‐canal medications.^3^ Calcium hydroxide was employed as the intracanal medication in the current case study.

Nowadays calcium hydroxide is commonly employed as an intracanal medication because of its wide variety of biological properties like antiseptic, anti‐exudative, and mineralization inducing properties.^3^, ^8^ Primary effects of calcium hydroxide has an impact on inflammatory tissue and epithelial cyst linings. This action of calcium hydroxide would support and cause periapical healing and stimulate osseous repair.^6^, ^9^

The most widely utilized and easily accessible pre‐mixed calcium hydroxide‐iodoform‐silicon‐oil pastes are called Metapex and Vitapex. It has antibacterial and bacteriostatic characteristics that aids in apexification and apexogenesis. Calcium hydroxide stimulates blast cells that promotes a procedure called apexogenesis and because of its high pH endotoxins from anaerobic bacteria are neutralized. Iodoform releases free iodine which eliminates root canal infection and also increases radiopacity for better visualization. So, it can be stated that iodoform has the bacteriostatic property. Silicone oil acts as lubricant which coats the root canal walls.^4^

To achieve periradicular area disinfection plus the healing process, several intracanal medicaments and irrigation strategies are being investigated for enhanced therapeutic results. A triple antibiotic paste (ciprofloxacin, metronidazole, and minocycline) are being employed recently. These antibacterial medications contribute to the disinfection of damaged root dentin. Ozone is highly biocompatible as well as a potent antioxidant having little adverse consequences. Investigation suggests that it can efficiently react and eliminate microbes in the root canal network. Being an antibacterial agent, ozone is 1.5 times more efficient than chlorine on a range of microorganisms, suggesting its great oxidative capability.^10^

Hence nonsurgical healing of periapical infection accompanied by the mixture of of root canal therapy and intracanal medicament provides a favorable clinical and radiographic response. But it is always mandatory to have follow‐ups of periapical lesion progressively as time passes after endodontic approach without surgery. Surgical intervention should only be considered when approach without surgery or retreatment procedures are not likely to provide the desired end result.^6^

The periapical lesion and extraoral sinus tract in the above case healed after the endodontic therapy without surgery. Peripaical regions have an enormous wealth of blood vessels, lymphatic, and undifferentiated mesenchymal cells, all of which promote rapid regeneration.^3^

## 7 CONCLUSION

In the present case report, endodontic approach without surgery using the correct cleaning, decontamination, filling of the root canal framework, and administration of intracanal medication such calcium hydroxide produced beneficial outcomes in aiding the recovery of lesions in the periapical region. No matter how big the area of damage is, endodontic therapy without surgery should always be tried for managing periapical lesions. Also it is necessary to have a follow‐ups for better prognosis.

## AUTHOR CONTRIBUTIONS

**Nitin R. Rao:** Data curation; investigation; methodology; resources; writing – original draft. **Abdul Mujeeb:** Investigation; methodology; supervision; writing – original draft; writing – review and editing. **Aishwarya A. Kottur:** Data curation; investigation; validation; writing – original draft. **Ankita Mathur:** Writing – original draft; writing – review and editing. **Niher Tabassum Siddiqua Snigdha:** Writing – original draft; writing – review and editing. **Mohmed Isaqali Karobari:** Conceptualization; methodology; supervision; writing – original draft; writing – review and editing.

## FUNDING INFORMATION

None.

## CONFLICT OF INTEREST STATEMENT

All authors disclose that there is no actual or potential conflict of interest including any financial, personal or other relationships with other people or organizations.

## CONSENT

A written informed consent was obtained from the patient to publish this report in accordance with the journal's patient consent policy.

## Data Availability

The (clinical pictures and radiographs) data used to support the findings of this study are included within the article.

## References

[1] Karamifar K , Tondari A , Saghiri MA . Endodontic periapical lesion: an overview on the etiology, diagnosis and current treatment modalities. Eur Endod J. 2020;5(2):54‐67. 32766513 10.14744/eej.2020.42714 PMC7398993

[2] Tekwani RA , Nanda Z , Rudagi K , Reddy KK , Deore R , Fotani S . Nonsurgical Management of an Extraoral Sinus Tract of endodontic origin: a case report. J Oper Dent Endod. 2019;4(1):55‐56.

[3] Kumar BS , Rangareddy MS , Karteek BS , Reddy CL , Wahed MA . Non‐surgical endodontic approach for Management of Periapical Lesions with 6 months follow up: a case series. Eur J Mol Clin Med. 2021;8(2):1524‐1531.

[4] Al Khasawnah Q , Hassan F , Malhan D , et al. Nonsurgical clinical management of periapical lesions using calcium hydroxide‐iodoform‐silicon‐oil paste. Biomed Res Int. 2018;12:8198795. 10.1155/2018/8198795 PMC5829310 29619378

[5] Bhaskar SN . Nonsurgical resolution of radicular cysts. Oral Surg Oral Med Oral Pathol. 1972;34(3):458‐468. 4505760 10.1016/0030-4220(72)90325-8

[6] Gupta A , Duhan J , Hans S , Goyal V , Bala S . Non surgical management of large periapical lesions of endodontic origin: a case series. JOHCD. 2014;8(3):172‐175.

[7] Kumar KS , Subbiya A , Vivekanandhan P , Prakash V , Tamilselvi R . Management of an endodontic infection with an extra oral sinus tract in a single visit: a case report. J Clin Diagn Res. 2013;7(6):1247. 23905152 10.7860/JCDR/2013/5369.3064 PMC3708247

[8] Soares JA , Brito‐Júnior M , Silveira FF , Nunes E , Santos SM . Favorable response of an extensive periapical lesion to root canal treatment. J Oral Sci. 2008;50(1):107‐111. 18403894 10.2334/josnusd.50.107

[9] Tronstad L , Andreasen JO , Hasselgren G , Kristerson L , Riis I . pH changes in dental tissues after root canal filling with calcium hydroxide. J Endod. 1981;7(1):17‐21. 6938618 10.1016/S0099-2399(81)80262-2

[10] Kriplani S , Sedani S , Mishra A , Umre U . Non‐surgical management of periapical lesions with the use of newer modalities in adjunct to the conventional: a case series. Cureus. 2024;16(3):1‐5. 10.7759/cureus.57314 PMC11059846 38690465

